# Pediatric Allergic Fungal Rhinosinusitis: Does Age Make a Difference?

**DOI:** 10.7759/cureus.30984

**Published:** 2022-11-01

**Authors:** Hanin A Alamoudi, Samaher Alzabidi, Afnan F Bukhari, Faisal Zawawi

**Affiliations:** 1 Otolaryngology - Head & Neck Surgery, King Abdulaziz University, Jeddah, SAU; 2 Otolaryngology - Head and Neck Surgery, King Abdulaziz University Hospital, Jeddah, SAU

**Keywords:** pediatric, sinus surgery, children, rhinosinusitis, allergic fungal

## Abstract

Background

Allergic fungal rhinosinusitis (AFRS) is a hypersensitive response to fungi within the sinus cavity. Children represent a challenging group of patients with sinonasal disorders, as their sinus anatomy is not fully developed. This study aimed to determine the various clinical manifestations and management outcomes in children with AFRS.

Methods

A retrospective chart review of children who underwent sinus surgery for AFRS at a tertiary healthcare center between 2005 and 2021 was performed. Demographics, clinical manifestations, radiological and laboratory results, treatment regimens, complications, and recurrence rates were collected. Subanalysis was performed based on age at first surgery: group A (<13 years) and group B (≥13 years).

Results

Overall, 35 children underwent sinus surgery for AFRS during the study period. The mean patient age at the time of surgery was 14 years. Bilaterality was present in 15/35 (42.9%) patients and anosmia in 12/35 (34.3%). Polyps on examination were present in 31/35 (88.6%) patients and proptosis in 8/35 (22.9%). Sub-analysis revealed that group A showed less bilateral disease (11.4%) than group B (31.4%) and a lower Lund-Mackay score (median=11.50 and 17, respectively, p=0.002).

Conclusion

Age at surgery did not have an impact on the outcome. A high index of suspicion should be exercised when dealing with children with sinonasal symptoms that do not respond to routine treatment and should be investigated for chronic sinusitis.

## Introduction

Allergic fungal rhinosinusitis (AFRS) is a hypersensitive response to fungi within the sinus cavity. Approximately 5%-10% of adults with chronic sinusitis require surgery [1]. Bent and Kuhn reported a case series of 15 patients and suggested the currently used diagnostic criteria [2].

The incidence of AFRS has a geographical distribution pattern, with most cases occurring in high-humidity areas around the world [3-5]. Most patients initially present with gradual nasal obstruction, nasal discharge, anosmia, and headaches [6]. Along with the indolent nature of the disease, patients may develop facial dysmorphic features, such as proptosis and telecanthus [3], which can eventually lead to diplopia, visual field cuts, and acute vision loss [6-9]. Patients have also reported that sinus symptoms recur despite multiple surgeries and prolonged medical therapy [10].

The management of AFRS includes both surgical and medical therapies. Surgery is followed by adjuvant therapy, which is the gold standard of treatment. Adjuvant therapy consists of topical and oral steroids along with immunotherapy [11].

The pediatric population represents a challenging and interesting group of patients with sinonasal disorders. Depending on their age, some of their sinuses are not fully developed, and their smaller nasal anatomy represents a unique challenge for endoscopic sinonasal surgeries.

This study aimed to determine the various clinical manifestations, radiological findings, medical and surgical management, and outcomes of children with AFRS and to investigate the differences in presentation and outcome between young children and teenagers.

## Materials and methods

After receiving ethical approval from the institutional review board, we retrospectively reviewed the medical records of pediatric patients who attended the otolaryngology-head and neck surgery clinic at a tertiary healthcare center. The study included all patients who underwent sinus surgery between 2005 and 2021. 

Patients were included if they met Bent and Kuhn’s criteria, based on the data present at the time of review. Bent and Kuhn’s criteria were followed, except for unilaterality, due to various new publications that have discussed the presence of bilateral manifestation of AFRS [12-14]. We excluded patients aged ≥19 years.

Information obtained from medical records included demographics and clinical presentation, including nasal, orbital, and neural symptoms and signs. The presence of comorbidities, including asthma, primary ciliary dyskinesia, cystic fibrosis, and atopy, was documented. Laboratory results were obtained, including white blood cell (WBCs) differentials, absolute WBC count, total serum immunoglobulin E (IgE), and Phadiatop. The histopathological results of the surgical specimen were obtained, particularly for the presence of eosinophils, Charcot-Leyden crystals, fungal elements, such as hyphae, and necrotic inflammatory cellular debris. The cultures were reviewed to identify the causative fungi. Radiological images, including computed tomography (CT) in the axial and coronal planes of the nose and paranasal sinuses, were reviewed for all patients and evaluated as per Lund-Mackay scores, double density, calcification, and laterality of disease. The images were also assessed for orbital and skull base involvement. The authors defined orbital involvement as a breach of lamina papyracea with double-density opacification. Skull base involvement was defined as a breach of the skull base bone with double-density opacification.

Surgical complications, including bleeding, CSF leak, and vision changes, were reviewed, and the recurrence rate was noted. Treatment regimens were collected pre- and postoperatively for each patient. Subanalysis was then performed by dividing the patients into those under 13 years of age and 13 or older. The choice of the age of 13 was based on that most children by the age of 13 have had their sinuses pneumatized.

The chi-square test was used for categorical data and frequency for continuous data. The Mann-Whitney U test was used for the subanalysis of nonparametric groups. Significance was determined if the p-value was <0.05. Statistical analyses were conducted using IBM SPSS Statistics 26.0 (IBM Corp., Armonk, NY).

## Results

Demographics and clinical presentation

The medical records of 586 patients were reviewed, of which 35 met the inclusion criteria. The mean age was 14 years, and the sex distribution included 15 female and 20 male patients. (43% and 57% respectively).

The overall results of the 35 patients showed that 15 patients presented with bilateral symptoms (42.9%), only three had blurred vision (8.6%), one had rhinorrhea (2.9%), 12 presented with anosmia (34.3%), and six (17.1 %) had documented atopy.

On physical examination, almost all patients presented with nasal polyposis (n=31,88.6%), which was the most common presenting sign. Of the patients, 22 (62.9%) had a deviated nasal septum, eight (22.9 %) had proptosis, and nine (25.7 %) had fungal debris (Table 1).

**Table 1:** This table highlights the clinical presentation (symptoms and signs) in both groups

	Group A N(%)	Group B N(%)	p-value
Bilateral Symptoms	4 (33.3)	11 (47.8)	0.489
Anosmia	3 (25)	9 (39.1)	0.476
Change in vision	1 (8.3)	2 (8.7)	1.000
History of atopy	3 (25)	3 (13)	0.391
Polyps	10 (83.3)	21 (91.3)	0.594
Proptosis	3 (25)	5 (21.7)	1.000
Reduced eye motion	0 (0.0)	2 (8.7)	0.536

Management 

The surgical nasal specimens sent for histopathology showed fungal hyphae and eosinophils in 21 samples (60.0%), 11 specimens exhibited Charcot-Leyden crystals (31.4%), and 24 specimens showed necrotic inflammatory cellular debris (68.6%). Twenty-seven cultures were positive for Aspergillus (A.) spp., with the following subtypes: 20 patients (57.1%) for Aspergillus flavus, three patients (8.6%) for A. niger, two patients (5.7%) for A. fumigatus, one patient (2.9%) for A. nidulans, and one (2.9%) for A. terreus.

IgE levels were measured in 23 (65.7%) of the 35 patients, with a mean of 1,273.3 IU/ml (normal range 0-199 IU/ml).

Radiologically, unilateral disease was more common than bilateral disease (54.3% vs. 42.9%). Overall, 17 patients had skull base involvement (48.6%), and more than two-thirds (26) had orbital involvement (74.3%). The mean Lund-Mackay score was 15.29.

The Mann-Whitney U test was performed to compare serum IgE levels with skull/orbital involvement, which was statistically insignificant. (U=41.000, p=0.906).

Lund-Mackay scores were divided into two groups: <12 and ≥13; another Mann-Whitney test was performed between them and IgE, which resulted in insignificance (U= 57.000, p= 0.920).

All the patients underwent endoscopic surgery (100%). Regarding postoperative complications, only one patient had a CSF leak (2.9%), one patient had bleeding (2.9%), and no change in vision was noted in any patient. Eight patients experienced disease recurrence (22.9%). The average length of hospitalization was two days. Medications prescribed for patients in the pre- and postoperative periods are listed in Table 2.

**Table 2:** This table lists the medical management that was provided to the patients both pre- and postoperatively

Medication	Pre-operative N (%)	Post-operative N (%)
Saline	12 (34.3)	13 (37.1)
Topical steroids	20 (57.1)	21 (60.0)
Budesonide rinses	1 (2.9)	14 (40.0)
Systemic steroids	24 (68.6)	29 (82.9)
Antibiotics	10 (28.6)	34 (97.1)
Antihistamine	4 (11.4)	5 (14.3)
itraconazole	0 (0.0)	1 (2.9)

Subanalysis 

Based on the age at first surgery, the patients were divided into group A (<13 years) and group B (≥13 years). In group A, there were 12 patients and in group B, there were 23 patients.

The median age of the patients in groups A and B was 10 and 16 years, respectively. Patients in group B complained mainly of bilateral nasal symptoms in the form of congestion, discharge, obstruction, and facial pain (31.4%) compared to those in group A (11.4%). Anosmia was more frequent in group B than in group A (25.7% vs. 8.6 %).

Moreover, group B had a higher incidence of polyps than group A (60.0% vs. 28.6%). Also, group B more frequently presented with deviated nasal septa (42.9% vs. 20.0%). Proptosis incidence was similar in both groups (Group = 8.6% and group B = 14.3%).

Serum IgE values were found to be insignificant but higher in group B, with a median of 985.1 IU/ml, compared to that of group A, with a median of 672.0 IU/ml. (U=54.000, p=0.764). Absolute eosinophil counts were not significantly different between the two groups (U=101.500, p=0.456) (Table 3).

**Table 3:** This table compares the radiological findings between both groups

	Group A (%)	Group B (%)	P-value
Double density	n= 12 (100)	n= 23 (100)	-
Calcification	n= 11 (91.7)	n= 17 (73.9)	0.380
Skull base involvement	n= 4 (33.3)	n= 13 (56.5)	0.289
Orbital involvement	n= 8 (66.7)	n= 18 (78.3)	0.685

On radiological assessment, bilateral disease was more common in group B (56.5% compared to 16.7% in group A). The majority of patients in group B had evidence of skull base involvement (n=13, 56.5%) compared to group A (n=4, 33.3%) (p=0.289). Orbital involvement was present in the majority of patients in both groups with slight predominant in group B (78.3%) compared to 66.7% in group A. The median Lund-Mackay score in group A was 11.50 while it was 17.00 in group B and was statistically significant (p=0.002, U=50.500). The average length of hospitalization was one day in both groups (U=111.000, p=0.317) (Table 4).

**Table 4:** This table compares the complication rates in both groups

	Group A N(%)	Group B N(%)	p-value
Bleeding	0 (0.0)	1 (4.3)	0.76
CSF leak	0 (0.0)	1 (4.3)	0.78
Vision change	0 (0.0)	0 (0.0)	-
Disease recurrence	3 (25)	5 (21.7)	0.59
Median Hospital stay	2	2	-

## Discussion

Regions with high humidity influence the AFRS incidence; therefore, it is not uncommon in children and often requires a combined medical and surgical approach [15,16]. The nature of AFRS tends to be gradual in onset and slowly progressive; therefore, it is crucial to identify children with AFRS, as they may develop facial skeleton abnormalities in the form of proptosis, telecanthus, and/or malar flattening [6-8].

Several studies have reported that AFRS presents as unilateral disease in children [7,17]; on the contrary, there was more bilateral presentation in our population (43.8%), similar to Kaur et al. (45.71%) [18].

The clinical presentation in this study mainly included nasal obstruction, congestion, anosmia, facial pain, and clear rhinorrhea, in accordance with the literature [6,7,19,20]. Proptosis was found in 25.0% of the children in our study. Similarly, Campbell et al. showed that 50% had proptosis [21]. However, the presentation differs from adults; pediatric patients were noted to have more facial dysmorphism, mostly in the form of proptosis [7]. McClay et al. reported an incidence of 42% in children compared with 10% in adults [7], and Gupta et al. reported that 60% of children presented with proptosis compared with 20% in adults [22]. This difference may be attributed to the fact that the facial skeleton continues to grow and completes its growth by adolescence [7].

Nasal polyposis was the initial presentation in most children (87.5%). Its presence should always lead to the suspicion of cystic fibrosis or any ciliary disorders and should always be kept as a differential [17,23,24].

Serum IgE levels were considerably high in a majority of our patients (53.1%) (>200 IU/mL), with a mean of 960.05 IU/ml, nearly similar to 51.5% [22]. Moreover, high IgE levels have been linked to pediatric patients, suggesting a higher fungal load with increased sensitivity to fungal antigens.

Aspergillus is primarily responsible for causing AFRS; however, other fungi may also cause the disease [21]. In our study population, A. flavus was the most commonly identified subtype, similar to the results of Patro et al. [17].

Regarding radiological assessment, a large inter-institutional study concentrating mainly on the laterality of fungal disease concluded that bilateral disease was more common than unilateral disease [21]. Historically, AFRS has been described as unilateral disease based on Bent and Kuhn’s case series of 15 patients, in which 13 had unilateral disease [2]. This is interestingly similar to our findings, with a unilateral predominance in children (46.9%).

In terms of orbital and skull base involvement, multiple studies have reported that the incidence of bone erosion falls within a range of 20% to 90% [25]. In our population, it was detected in 78.1% of the patients, which is comparable to the results of Gupta et al., who reported bony erosion in 88.0% of the children [16]. Additionally, Ghegan et al. noted skull or orbital involvement in 56.0% of cases, and Nussenbaum et al. reported bony erosions in 20.0% of cases [25,26].

Skull base extension in this pediatric cohort was noted in 50.0% of the patients while Liu et al. reported skull base extension in 38.1% of patients [27]. Orbital involvement in this study was detected in 71.9% of patients, whereas Liu et al. reported 28.6% of cases of orbital involvement [27]. Calcifications were demonstrated in 78.1% of the patients, and the double-density sign was also seen in all of our patients on CT scan (100%) while another study showed the double-density sign in only 78.7% of cases [28].

Recurrence is the main issue in AFRS and can be as high as 100% if surgery is not followed by postoperative medical therapy [8]; therefore, a combined surgical and medical treatment strategy has been developed [1,14]. Adequate sinus surgery is necessary and is the first step in its management. All patients underwent endoscopic surgery to ensure complete surgical removal of allergic mucin and debris [19,29]. Administration of nasal saline irrigation along with intranasal and oral corticosteroids has shown favorable effects in the postoperative period [27]. Administering local and systemic steroids preoperatively is an accepted practice to reduce inflammation [20,30]. Some support the use of steroids in combination with antibiotics to decrease the risk of concurrent bacterial infection [11,19,20,28]. Our patients were administered all three in the pre- and postoperative periods in addition to Pulmicort and antihistamine. One of the patients received antifungal therapy (itraconazole), which has been reported in the literature to treat recurrence successfully with minimal side effects [27,31].

Regarding the length of hospitalization, as a referral center, the hospital receives many patients from far distances, and despite physicians wishing to discharge patients on the same day of surgery, it is not feasible due to social reasons.

The main finding of this study is that older children (group B) had more bilateral symptoms than those in group A. Thus, this could indicate the possibility of the disease being bilateral in nature and to always keep AFRS at the top of the differential diagnosis.

This study had the limitations of small sample size and lack of long-term follow-up. Another limitation is the retrospective design, as not all patients underwent the same pre- and postoperative care in a standardized manner.

## Conclusions

The age of the child did not have a significant impact on clinical presentation or the outcome of management. Orbital and skull base involvement is common in children and care should be taken when managing children with AFRS.
